# Temporal Neural Network Framework Adaptation in Reconfigurable Intelligent Surface-Assisted Wireless Communication

**DOI:** 10.3390/s23052777

**Published:** 2023-03-03

**Authors:** Mohammad Abrar Shakil Sejan, Md Habibur Rahman, Md Abdul Aziz, Young-Hwan You, Hyoung-Kyu Song

**Affiliations:** 1 Department of Information and Communication Engineering, Sejong University, Seoul 05006, Republic of Korea; 2 Department of Convergence Engineering for Intelligent Drone, Sejong University, Seoul 05006, Republic of Korea; 3 Department of Computer Engineering, Sejong University, Seoul 05006, Republic of Korea

**Keywords:** temporal neural network, machine learning, reconfigurable intelligent surface, wireless communication

## Abstract

A reconfigurable intelligent surface (RIS) has potential for enhancing the performance of wireless communication. A RIS includes cheap passive elements, and the reflecting of signals can be controlled to a specific location of users. In addition, machine learning (ML) techniques are efficient in solving complex problems without explicit programming. Data-driven approaches are efficient in predicting the nature of any problem and can provide a desirable solution. In this paper, we propose a temporal convolutional network (TCN)-based model for RIS-based wireless communication. The proposed model consists of four TCN layers, one fully connected layer, one ReLU layer, and lastly a classification layer. In the input, we provide data in the form of complex numbers to map a specified label under QPSK and BPSK modulation. We consider 2×2 and 4×4 MIMO communication using one base station and two single-antenna users. We have considered three types of optimizers to evaluate the TCN model. For benchmarking, long short-term memory (LSTM) and without ML are compared. The simulation results are conducted in terms of the bit error rate and symbol error rate which show the effectiveness of the proposed TCN model.

## 1. Introduction

**Figure 1:**
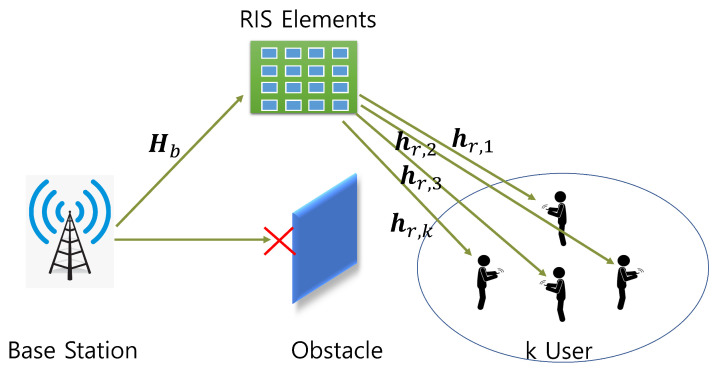
RIS working scenario for wireless communication system blockage.

The future wireless communication system has a high demand for a high data rate, high spectral efficiency, and uninterrupted service to the end user [1]. Fifth-generation (5G) and sixth-generation (6G) mobile communication provide a multi-gigabit communication system for the users [2]. However, the present technologies are struggling to provide high-speed data connections, and breakthrough technologies are being investigated to provide adequate support. Specifically, new solutions of new spectrum and low-energy consumable techniques along with a minimum hardware cost are desirable. Many new concepts and technologies are now being researched for solving wireless communication-related problems. Recently, a new paradigm for wireless communication was introduced as a reconfigurable intelligent surface (RIS) [3,4,5,6]. A RIS is a planar array of a large number of passive elements, where each element can change the phase shift of the incident signal independently [7]. Each element is a low-cost inexpensive surface of an electromagnetic material which can be controlled by a smart controller. This gives a unique opportunity not only to reflect but also to modify the shape of the reflected signal. Currently, RIS implementations include a conventional reflection array, software-defined meta-surfaces, and liquid crystals [8]. Figure 1 shows a typical application scenario for a RIS-based communication system. The original signal is blocked due to some obstacles and an alternative path is being created via RIS elements. The base station can serve the user in spite of the signal being blocked by obstacles. Usually, the RIS elements are mounted on a wall to reflect the signal to the user location.

Machine learning (ML) techniques are considered as a powerful tool for solving different complex problems without programming explicitly. Data-driven approaches can be useful in predicting the behavior of a system with high efficiency. This case features ML-related algorithms in a vast area of different research domains. ML-based algorithms are also deployed in wireless communication areas for performance improvement [9]. ML is broadly classified as supervised learning, unsupervised learning, and reinforcement learning. In supervised learning, a model is trained against an input–output mapping scenario. All the labeled data are provided to the system and the system learns the parameters depending on the corresponding labels. This technique is mostly used when training data are easily available. In unsupervised learning, there is no label associated with the data. Here, the model tries to learn the pattern in the data and tries to cluster the data into groups. Reinforcement learning tries to find a good policy from the feedback that it receives from the environment. ML was also introduced in RIS-based communications [10]. A deep learning-based approach was adopted in [11,12,13,14,15]. Unsupervised-based approaches were studied to solve different problems, such as the deployment of the base stations, user equipment clustering or association, detection of network state, aggregation of a dataset, and cancellation of interference [16,17,18,19]. In [20], the authors proposed a UL-CNN or sum rate maximization with an unsupervised approach. The authors in [21] also used an unsupervised learning approach for RIS-assisted industrial internet of things connectivity. Reinforcement learning was adopted in [22,23,24] for RIS-based communications.

Convolutional neural networks (CNN) can provide a superior performance in computer vision-related works. However, the structure of a CNN does not give optimal results in time series or sequence data classifications to recognize long-range patterns. To work with series data using a CNN, the authors in [25] proposed a temporal convolutional network (TCN). A TCN consists of 1-D convolutions of a large temporal receptive field with a few parameters as compared to other models. Previous studies showed that a TCN can provide outstanding results in speech signal processing [26,27,28], human action recognition [29], the charge estimation of Lithium-ion batteries [30], anomaly detection in IoT networks [31], and so on. However, to the best of our knowledge, a TCN has not been applied in wireless communication and data demodulation applications. This approach can be a promising solution in wireless communication as the received data can be represented in sequence. We can apply the sequence to label mapping using a TCN for regenerating bits at the receiver. Thus, we study the outcome of the TCN model for wireless communication scenarios for RIS applications.

The contributions of this study can be listed as follows:

- We propose a new TCN-based machine learning framework for recovering end-user data using RIS-based signal reception. We consider multiple user signals transmitted from a single base station reflected by a RIS.
- We evaluate the performance of the proposed model in terms of the bit error rate (BER) and symbol error rate (SER) with two different modulation schemes, such as quadrature phase-shift keying (QPSK) and binary phase-shift keying (BPSK).
- Different optimizers are employed to observe the performance variation for the model. In addition, a different system configuration is considered for testing the effectiveness of the TCN model.
- For benchmarking, long short-term memory (LSTM) and without ML are taken into account.

The rest of this paper is organized as follows. Section 2 represents the RIS-based communication scenario for MIMO communication, Section 3 describes the construction of the TCN model and the different features, Section 4 describes the simulation results of the RIS-enhanced communication demodulation system, and finally, Section 5 represents the conclusions.

## 2. RIS-Assisted Communication System Model

Figure 1 shows the conceptual idea of a typical RIS-based communication system. We have considered the downlink communication for a multi-antenna base station (BS) to *K* single-user equipment (UE). In the BS, there are *M* uniform planar array antennas and the RIS has *N* number of reflective elements. The signal received by the *k*th UE is a combined signal from BS to RIS and from RIS to UE. Thus, the receiver signal via RIS for the *k*th UE can be represented as follows [32]:

(1) $$y_{k}=h_{r,k}^{H}\Psi H_{b}x+n_{k},$$

where $H_{b}\in C^{N\times M}$ is the channel from BS to RIS, $h_{r,k}\in C^{1\times N}$ is the channel from RIS to the *k*th user, *x* is the transmitted symbol, and $n_{k}$ is the additive white Gaussain noise (AWGN) with $n_{k}\;\mathrm{CN}\left(0,\sigma_{k}^{2}\right)$. Ψ= diag $\left(c\right)\in C^{N\times N}$ is the diagonal matrix presenting the phase-shift values of the reflecting elements of RIS with $\left(c\right)=[\alpha_{1}e^{j\theta_{1}},\alpha_{2}e^{j\theta_{2}},...,\alpha_{N}e^{j\theta_{N}}$], where α∈[0,1] is the amplitude of the signal and $\theta_{n}\in \left[0,2\pi \right]$ is the phase-shift value. In this paper, the constant amplitude coefficient ($\alpha_{N}=1$) is assumed [33]. Next, the entire channel from the BS to the *k*th UE through RIS is represented by $h_{r,k}^{H}\Psi H_{b}\in C^{1\times M}$. Specifically, it is worth to note that the matrix Ψ= diag (c) is the diagonal matrix. Then, the aforementioned entire channel matrix $h_{r,k}^{H}\Psi H_{b}$ can be rewritten as follows [32]:

$$\begin{array}{cc} h_{2,k}^{H}\Psi H_{b}= & h_{2,u}^{H}\mathrm{diag}\left(c\right)H_{b}=c^{T}\mathrm{diag}\left(h_{2,k}^{H}\right)H_{b}\\ H_{k}= & \mathrm{diag}\left(h_{2,k}^{H}\right)H_{b}. \end{array}$$

For the mmWave communication, BS-RIS channel can be expressed as follows [34]:

(2) $$H_{b}=\sqrt{\frac{NM}{P_{b}}}\sum_{p=1}^{P_{b}}\delta_{p}a_{r}\left(\theta_{p},\gamma_{p}\right),a_{t}\left(\phi_{p}\right),$$

where $\delta_{p}$ is the complex gain with the associated *p*th path, $\theta_{p}\left(\gamma_{p}\right)$ is azimuth (angle) of angle of arrival (AoA), $\psi_{p}$ is the angle of departure (AoD), $a_{r}\left(\theta_{p},\gamma_{p}\right)$ is the steering vector associated with receiver, and $a_{t}\left(\phi_{p}\right)$ is the steering vector associated with transmitter. For a typical $M=M_{1}\times M_{2}$ UPA, the array response vector is written as follows:

(3) $$a\left(\theta ,\gamma \right)=\frac{1}{\sqrt{M}}\left[e^{-j2\pi d\sin\left(\theta \right)\cos\left(\gamma \right)m_{1}/\lambda }\right]⊗\left[e^{-j2\pi d\sin\left(\gamma \right)m_{2}/\lambda }\right],$$

where $m_{1}=\left[0,1,\ldots ,M_{1}-1\right]$, $m_{2}=\left[0,1,\ldots ,M_{2}-1\right]$, λ is the wavelength, *d* is the antenna spacing with d=λ/2. The channel between RIS and UE can be expressed as follows:

(4) $$h_{r,k}^{H}=\sqrt{\frac{N}{P_{r,k}}}\sum_{p_{2}=1}^{Pr,k}\delta_{p_{2}}a\left(\theta_{p_{2}},\gamma_{p_{2}}\right),$$

where $P_{r,k}$ is the number of paths between RIS and UE, $\delta_{p_{2}}$ is the complex gain including path loss, and $\theta_{p_{2}},\left(\gamma_{p_{2}}\right)$ is the azimuth (elevation) angle at RIS for the $p_{2}$th path.

## 3. Proposed TCN Model Architecture

Different types of sequence data modeling and forecasting are performed in deep learning approaches with LSTM and gated recurrent units (GRUs). CNNs are superior in computer vision applications, such as image classification, object detection, autonomous cars, and biometric authentication problems. However, in sequence classification and forecasting problems, CNN has drawbacks due to a lack of memory. TCN was proposed in [25], which modified the CNN architecture to adopt sequence data and time-series prediction.

For TCN, we consider one-dimensional (1-D) input sequence $X=\{x_{1},x_{2},\ldots ,x_{n}\}$ of features or data. We can predict some output as $Y=\{y_{1},y_{2},\ldots .y_{m}\}$. The sequence modeling can be written as f:X→Y. The input-to-output mapping can be written as follows:

(5) $$y_{1},y_{2},\ldots ,y_{m}=f\left(x_{1},x_{2},\ldots x_{n}\right).$$

**Figure 2:**
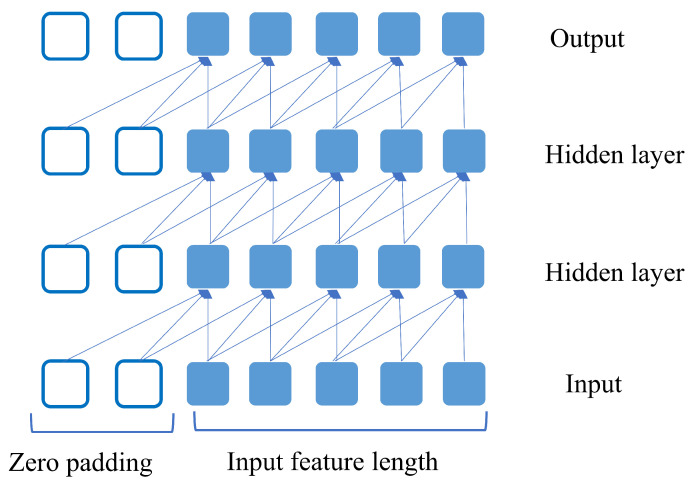
Example TCN with 4 layers where the number of input features is 5 and output is also 5.

**Figure 3:**
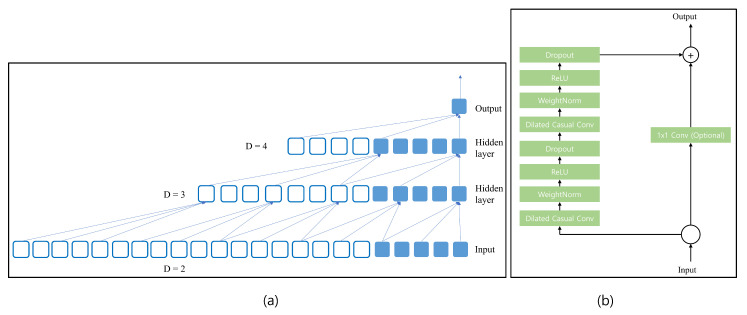
(**a**) Example of TCN with different dilation factors 2, 3, and 4 with kernel size 3, (**b**) different layers are present in the TCN block.

TCN workflow with the sequential data in each layer is shown in Figure 2. One of the characteristics of TCN is that it has an equal number of inputs and output sequences in each layer. This is ensured by adding zero padding to each layer shown in the white box of Figure 2. TCN uses casual convolution to stop the leakage of any information from the future to the past. This design can have a very long history size and can handle a network for which large size kernels are needed. To tackle this problem, dilation is introduced in TCN. For a 1-D sequence input $x\in R^{n}$ with a filter f:{0,1,….q−1}→R, the dilated convolution operation D for sequence *t* is written as follows:

(6) $$D\left(t\right)=\left(x{*}_{d}f\right)\left(t\right)=\sum_{i=0}^{q-1}f\left(i\right)\cdot x_{t-d\cdot i},$$

where *d* is the dilation factor, *q* is the filter size, and t−d·i is the direction of the past. Thus, dilation is considered as a fixed step between every two filter taps. Figure 3a shows an example of dilation factors with d=2,3,4. The receptive field for *n* layer convolutional network with kernel size *q* is as follows:

(7) r=1+n∗(q−1).

To complete the structure of TCN, the residual block is added for learning the identity mapping. If *x* is the input, then the output of the residual block is as follows:

(8) β(x)=F(x)+x.

In the case of TCN as shown in Figure 3b, the residual block consists of dilated causal convolution block, weight normalization block, rectified linear unit (ReLU), and dropout block. The same configuration is repeated two times, and after that, the input is added to the resulting value.

### 3.1. Dataset Generation

We consider the RIS channel for wireless communication, and the direct communication channel is not taken into account. Random data bits are generated and transmitted through the channel with AWGN. The parameters are listed in Table 1. QPSK and BPSK are adopted along with orthogonal frequency division multiplexing (OFDM). In the OFDM, the subcarrier length is 128, and the cyclic prefix is 32. The binary data elements are transformed into OFDM symbol and the symbol is assigned to a corresponding label. We consider two complex data sequences for training. One is before adding noise and the second is after adding noise. Each sequence has a real and imaginary part that is separated and assigned with a label. The data symbol for QPSK and BPSK before noise can be expressed as follows:

(9) $$\xi =R_{e}+I_{m}.$$

Again, the data symbol after adding noise can be expressed as:

(10) $$\xi_{n}=R_{e}+I_{m}.$$

Thus, one label can be mapped as the following values:

(11) $$\Upsilon =[\xi \;\xi_{n}].$$

For QPSK, each transmitting antenna can have 4 unique symbols. Thus, for two antennas that can represent $4^{2}=16$ combinations, each can express different labels. In addition, two user data are considered simultaneously which makes the input feature as follows:

(12) $$\Upsilon_{in}=\left[\xi \;\xi_{n}\;\xi \;\xi_{n}\right].$$

Similarly, for BPSK, each transmitting antenna can have 2 unique symbols. Thus, for two antennas that can represent $2^{2}=4$ combinations, each can express different labels. Two user data can be expressed as follows:

(13) $$\Upsilon_{in}=\left[\xi \;\xi_{n}\right].$$

### 3.2. Model Description

**Figure 4:**
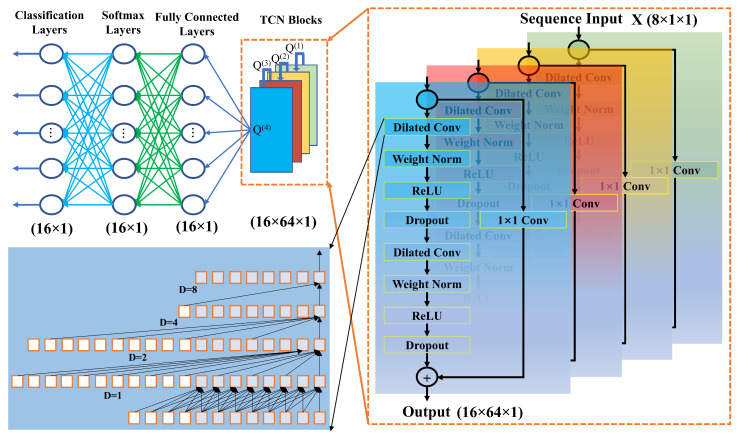
Block diagram of the proposed TCN-based demodulation technique at the receiver. The received input sequence is fed into the TCN network as (8×1×1) features and four TCN blocks employ $Q^{\left(1\right)},Q^{\left(2\right)},Q^{\left(3\right)},Q^{\left(4\right)}$; for the dilated convolution layer, we have used four dilation values (1, 2, 4, 8), kernel size 6, and 64 filters; the final output of $Q^{\left(4\right)}$ is connected to a fully connected layer with dimension (16×64×1), followed by a softmax layer with dimension (16×1), and a classification layer with dimension (16×1).

In this section, we describe the proposed model using TCN. In the proposed system, we map sequence to label for predicting communication data. The proposed TCN-based prediction model is shown in Figure 4. Each of the sequence data has eight samples $X=\{x_{1},x_{2},\ldots .,x_{8}\}$ for QPSK and each sequence has four samples $X=\{x_{1},x_{2},x_{3},x_{4}\}$ for BPSK. The TCN-based model is placed on the receiver side for generating demodulated data label $y_{k}$. In the beginning, the input layer receives the sequence data which are complex, including real and imaginary parts via a wireless channel. Thus, the input features of the convolutional layer are 8 and 4. TCN blocks are employed for processing, connected in feedforward propagation. As depicted in Figure 4, four dilation factors were used in the convolution dilation layers 1, 2, 4, and 8. Next, the output of the first TCN block $Q^{\left(1\right)}$ is connected to the input of the second block. The second block produces output $Q^{\left(2\right)}$ which is then connected to the input of the third block. Again, the output of the third block $Q^{\left(3\right)}$ is then connected to the fourth block of TCN. The output of the fourth block $Q^{\left(4\right)}$ is connected to a fully connected layer. Each TCN block is connected in a serial way and the last output is connected to a fully connected layer and is mathematically expressed as follows:

(14) $$F^{\left(1\right)}=Q^{\left(4\right)}*W+b,$$

where *W* is the weight matrix and *b* is the bias vector. The fully connected layer $F^{\left(1\right)}$ is connected to Softmax layer as follows:

(15) $$S^{\left(1\right)}=Z^{\left(1\right)}\left(F^{\left(1\right)}\right),$$

(16) $$Z^{\left(1\right)}\left(\vec{a}\right)=\frac{exp^{a_{j}}}{\sum_{k}exp^{a_{k}}},$$

where $Z^{\left(1\right)}$ is the Softmax function. Finally, the output of the classification layer is as follows:

(17) $$y^{\left(i\right)}=f\left(S^{\left(1\right)}\right),$$

where $y^{\left(i\right)}$ is the ith predicted label of the input feature.

## 4. Simulation Results

In this section, the outcome of the proposed model based on RIS-assisted communication is presented. The simulation results are obtained under the hardware system environment of Windows 10 pro with a graphics processing unit (GPU). The programming is performed with MATLAB software with the help of the Deep Learning Toolbox^TM^. The proposed TCN model simulation parameters are shown in Table 2. For the simulation, the BS antenna M=2, RIS reflecting elements N=32×16, and number of UEs K=2 are considered for the BS-RIS-UE configuration. The pathways between the BS to RIS and RIS to UE are considered to be $P_{b}=2$ and $P_{r,k}=2$, respectively. For the current study, we considered a Rician *K*-factor of 15 dB and it was assumed that all of the communication network parameters are static, that all the UEs are in the static position, and the distance from the BS to RIS and the RIS to UE is not changed.

**Figure 5:**
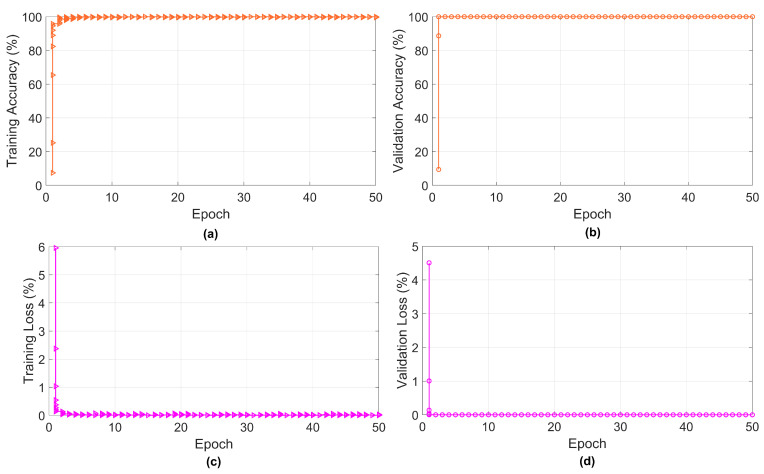
Training progress of proposed TCN model: (**a**) training accuracy with 50 epochs, (**b**) validation accuracy with 50 epochs, (**c**) validation accuracy with 50 epochs, and (**d**) validation loss with 50 epochs.

To generate the label and corresponding dataset for training the proposed TCN network, two modulation schemes, QPSK and BPSK, are considered. In addition, three optimization algorithms (Adam, stochastic gradient descent with momentum (SGDm), and root mean squared propagation (RMSprop)) are employed during the training of the TCN model. A total of 256,000 dataset samples are generated for training the model, where 80% and 20% of the data are divided for the training and validation of the proposed TCN. At the time of dataset generation, a 30 dB SNR is considered. Then, the TCN network is configured with the training parameters to successfully run it for learning the generated data. To minimize the loss function during training, the above-mentioned optimization algorithms are used. For optimal learning, the learning rate of 0.01 is used. The proposed model has completed 100 epochs for satisfactory learning, and at this stage, the validation accuracy achieved is about 99.97%. The training validation summary of the QPSK with 50 epochs taken is shown in Figure 5a–d where the different graphs indicate the training accuracy and loss and validation accuracy and loss. In this work, for receiving, the multiuser is considered. Therefore, to eliminate the inter-user interference (IUI) on the terminal side, the zero-forcing (ZF) precoder [35] is taken into account. The simulation results under the SER and BER performance with respect to different SNR levels are performed to test the efficiency of the proposed TCN model for the RIS-assisted communication system.

**Figure 6:**
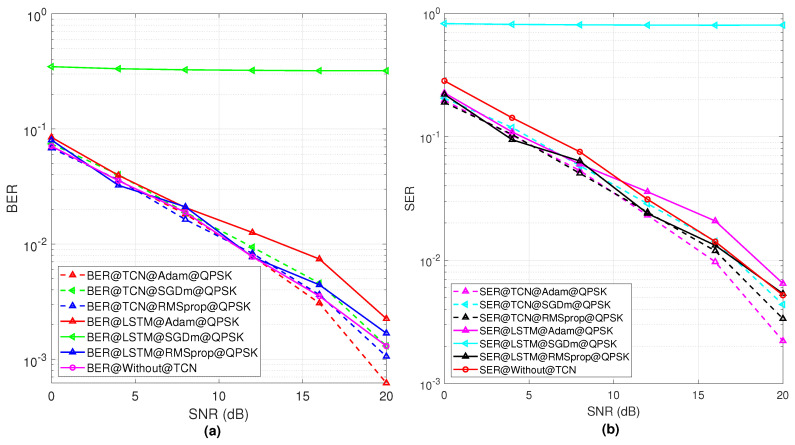
(**a**) The BER results with respect to SNR of different optimizers used for TCN model using QPSK modulation, (**b**) represents SER performance with respect to SNR values for the QPSK modulation scheme for different optimizers.

**Figure 7:**
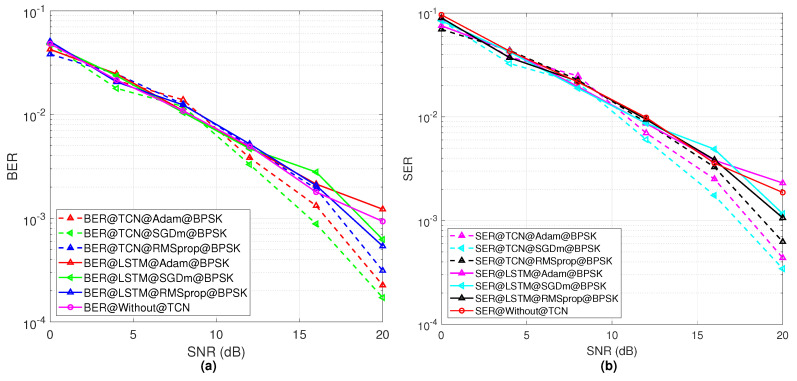
(**a**) The BER results for different optimizers with respect to SNR values for the BPSK modulation, (**b**) SER performance with respect to SNR values for the BPSK modulation scheme for different optimizers.

To test the performance of the proposed TCN, we have compared the BER and SER with the time-series LSTM model as well as the without TCN model. The calculation of the BER and SER at the receiver side is as follows: (1) the error rate of the BER describes the incorrect demodulated bit at the receiver and (2) the incorrect categorization of the received symbol means the error rate for the SER. Thus, in the simulation results, the BER and SER of the BPSK are different for the proposed system. In addition, the simulation results of the BER and SER for the BPSK and QPSK are taken over the AWGN. In this study, the application of the TCN is the pilot project for testing the RIS-based environment and further investigation is needed for the performance improvement. In the future, the TCN model will be tuned with proper parameters for obtaining a more optimal performance. Figure 6 shows the BER and SER performance of the proposed TCN model with consideration of the QPSK modulation. Figure 6a,b show the performance comparison with the proposed TCN, LSTM model, and without TCN model in terms of the BER and SER results. The results are performed by comparison of the three optimizers, Adam, SGDm, and RMSprop. From Figure 6a, it is shown that the performance of the BER with the Adam optimizer achieves better results compared to the two others. Next, the RMSprop optimizer as compared to the SGDm optimizer achieves a good BER. The overall error trend of the SER follows the BER trend which is shown in Figure 6b. It is shown that the Adam optimizer provides a better SER performance compared to RMSprop and SGDm. Again, it is shown from Figure 6b that RMSprop has a better performance than SDGm. From Figure 6 and Figure 7, it is observed that the proposed TCN model outperforms the LSTM and without TCN model in terms of the SER and BER simulation results with different optimization algorithms. In addition, it is noticeable that, with SGDm optimization, the LSTM model performance is very poor compared to the proposed TCN with the same simulation parameters.

On the other hand, Figure 7 represents the performance of the BER and SER of the proposed TCN model with consideration of the BPSK modulation. Figure 7a,b show the performance comparison with the proposed TCN, LSTM model, and without TCN model in terms of the BER and SER results. In this case, it is shown from Figure 7a that SGDm has a better performance as compared to the Adam and RMSprop optimizers. When fewer constellation points in the modulation scheme are used, SGDm can decode the received bits successfully. After that, Adam has the second highest BER performance and SGDm gives the worst performance in the case of the BER. Figure 7b shows the SER performance of the three optimizers, and a similar trend is shown like the BER results. In addition, compared with the LSTM and without TCN method, the proposed TCN-based model provides a better performance in terms of different SNR values.

**Figure 8:**
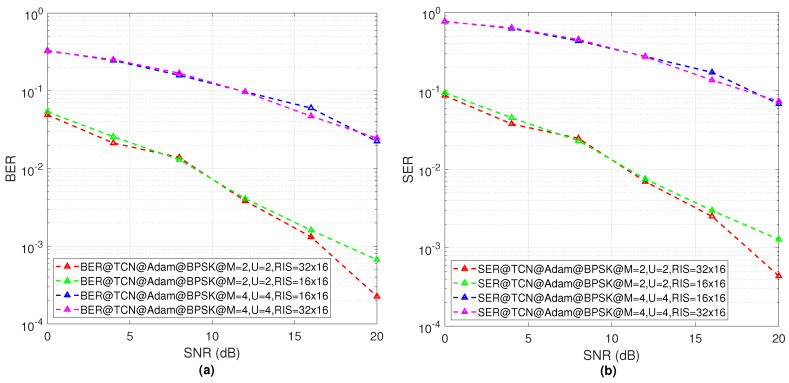
(**a**) The BER results of the proposed TCN model effectiveness for different system configurations, (**b**) SER results of the proposed TCN model effectiveness for different system configurations.

Figure 8a shows the BER result for the 2 × 2 MIMO with 256 and 512 RIS elements and the 4 × 4 MIMO with 256 and 512 RIS elements. For the 2 × 2 MIMO, the BER performance is improved according to the number of RIS elements. Again, for the 4 × 4 increasing RIS elements number, the BER performance has no significant changes for different SNRs. Figure 8b shows the SER performance for the 2 × 2 MIMO with 256 and 512 RIS elements and 4 × 4 MIMO with 256 and 512 RIS elements. For the 2 × 2 increasing RIS elements number, the SER performance is improved. However, for the 4 × 4 MIMO, the SER performance has no significant changes for different SNRs according to the number of RIS elements.

## 5. Conclusions

In this paper, for the first time, we proposed an ML-based TCN model for a RIS-assisted MIMO communication system. The goal of the proposed system is to analyze the implemented TCN model with a RIS channel for the observation of the BER and SER by changing the different optimizers during the training of the model. The effectiveness of the proposed model is analyzed with two different modulation schemes. The Adam, SGDm, and RMSprop optimizers are employed for loss function minimization. The simulation results of the proposed TCN model represented that the performance of the BER and SER with respect to different SNR values achieved satisfactory results. Thus, the TCN model can be a new candidate for wireless communication systems. In the future, the proposed TCN model can be tuned with different parameters and applied to more complex scenarios.

## Figures and Tables

**Table 1:** Dataset generation parameters.

Parameters	Value
Modulation	BPSK, QPSK
FFT size	128
Transmitter antenna	2
Receiver antenna	1
Number of users	2
Number of RIS elements	512 (32 × 16)
Number of paths from BS to RIS	2
Number of paths from RIS to UE	2
Noise	AWGN

**Table 2:** Model training parameters.

Parameters	Value
Max epochs	100
Learning rate	0.01
Dilation factors	1, 2, 4, 8
Number of filters	64
Dropout factor	50%
Input features	8
Number of output class	16
Kernel size	5 × 5
Optimizer	Adam, SGDm, RMSprop

## Data Availability

Not applicable.
